# A new beginning…

**DOI:** 10.4103/0972-3919.63588

**Published:** 2010

Dear Readers,

It gives us immense pleasure in presenting to you the current issue of Indian Journal of Nuclear Medicine (IJNM). We admit that IJNM is passing through a tough phase of its existence. Our new editorial team is committed to revive and regularize it. Our goal, primarily, would be to bring out its issues on time and revive its academic and scientific content.

The IJNM, similar to other journals, represents the intellectual and scientific mind set of a vibrant professional society. However, for many years, I have observed a sea of change in the intellectual and scientific culture of our society. We love to speak in seminars and conferences, and fight by all means to win a prime time slot in the conference proceedings. It must be providing an immense boost to the professional ego and an increase in the energy / adrenaline levels of the presenter. However, its impact on the audience remains as transient as the time period of the professional talk, varying from 10 to 30 minutes maximum. Even the audience shows their apathy by their thin attendance in large auditoriums.

The impact of scientific writing is everlasting. You can refer to it in the hour of need, update it with more experiences, and change it based on the current evidence. The written script is always open for scrutiny, which makes it a viable medium for interaction. Scientific literature is evidence-based rather than eminence-based. I have seen many members of the Society of Nuclear Medicine (SNM), India, keeping back issues of IJNM in hard-bound form, in book racks and departmental libraries as proud owners. We need to derive inspiration from such readers and inculcate the culture of scientific writing in our society.

We appeal to healthcare companies engaged in nuclear medicine and allied products, to support the scientific writing culture in our society. You will find this medium more economical in promoting your products and supporting genuine science.

We will be adding few valuable learning tools such as, 'Technology Update', 'Continuing Educational Activity', and 'Pictorial Essay' to the next issue of IJNM. Seeing the rapid growth of positron emission tomography-computed tomography (PET-CT), we need to prepare ourselves and the students for new challenges of fusion imaging. We are introducing a new column entitled 'Teaching File — Radiology for Nuclear Physicians'.

The current issue of IJNM will make us feel that we, as a society, are academically 'ALIVE'. We are committed to better editions in the future. We need your active support in the form of scientific writings, valuable suggestions, and comments in the 'Letter to Editor' column.

Yours in SNM,


Anshu Rajnish Sharma
Editor-in-Chief,
Bio-imaging Unit, Tata Memorial Hospital,
Parel, Mumbai - 400 012, India.
E-mail: editor@ijnm.in


